# Evidence for Detrimental Cross Interactions between Reactive Oxygen and Nitrogen Species in Leber's Hereditary Optic Neuropathy Cells

**DOI:** 10.1155/2016/3187560

**Published:** 2015-12-31

**Authors:** Micol Falabella, Elena Forte, Maria Chiara Magnifico, Paolo Santini, Marzia Arese, Alessandro Giuffrè, Kristina Radić, Luciana Chessa, Giulia Coarelli, Maria Chiara Buscarinu, Rosella Mechelli, Marco Salvetti, Paolo Sarti

**Affiliations:** ^1^Department of Biochemical Sciences and Istituto Pasteur-Fondazione Cenci Bolognetti, Sapienza University of Rome, Italy; ^2^CNR Institute of Molecular Biology and Pathology, 00185 Rome, Italy; ^3^Faculty of Pharmacy and Biochemistry, University of Zagreb, 10000 Zagreb, Croatia; ^4^Department of Clinical and Molecular Medicine, Sapienza University of Rome, 00185 Rome, Italy; ^5^Centre for Experimental Neurological Therapies, S. Andrea Hospital-Site, Department of Neuroscience, Mental Health and Sensory Organs (NESMOS), Sapienza University of Rome, 00185 Rome, Italy

## Abstract

Here we have collected evidence suggesting that chronic changes in the NO homeostasis and the rise of reactive oxygen species bioavailability can contribute to cell dysfunction in Leber's hereditary optic neuropathy (LHON) patients. We report that peripheral blood mononuclear cells (PBMCs), derived from a female LHON patient with bilateral reduced vision and carrying the pathogenic mutation 11778/ND4, display increased levels of reactive oxygen species (ROS) and reactive nitrogen species (RNS), as revealed by flow cytometry, fluorometric measurements of nitrite/nitrate, and 3-nitrotyrosine immunodetection. Moreover, viability assays with the tetrazolium dye MTT showed that lymphoblasts from the same patient are more sensitive to prolonged NO exposure, leading to cell death. Taken together these findings suggest that oxidative and nitrosative stress cooperatively play an important role in driving LHON pathology when excess NO remains available over time in the cell environment.

## 1. Introduction

Leber's hereditary optic neuropathy (LHON) is a mitochondrial disorder leading to severe visual impairment, due to retinal ganglion cells (RGCs) death and atrophy with demyelination of the optic nerve [1]. The primary cause of the disease is a mitochondrial genome (mtDNA) mutation leading to a single amino acid substitution in one of the mitochondrially encoded subunits of NADH:ubiquinone oxidoreductase, complex I of the electron transport chain (ETC). The most common mutations are at positions 11778/ND4, 3460/ND1, and 14484/ND6 [2, 3].

LHON pathology generally occurs in the second or third decade of life and affects predominantly males [4]. The only clinically relevant phenotype in most of the patients is RGCs loss, seldom accompanied by other complicating neurological disorders, such as dystonia, multiple sclerosis- (MS-) like illness parkinsonism, cerebellar ataxia, and myoclonus [5], pointing to a diffused mitochondrial energetic failure. Interestingly, a higher risk of developing MS in women with clinically established LHON has been reported [6–8]. Rarely other phenotypes have been described, such as chronic renal failure [9, 10], involving as common feature tissues/cells that are exquisitely energy dependent and require adequate supply of reducing substrates and O_2_ to sustain mitochondrial adenosine-5′-triphosphate (ATP) production.

All LHON mutations induce an impairment of mitochondrial function. A decline in complex I-sustained cell respiration and ATP production has been reported in assays performed on isolated mitochondria derived from muscle, Epstein-Barr Virus- (EBV-) transformed leukocytes, peripheral blood mononuclear cells (PBMCs), and cybrids [11–15]. Based on these specific defects, experiments carried out on patient's tissues and cell models of the disease showed an overproduction of reactive oxygen species (ROS) and an increased propensity to apoptotic cell death [16]. Nevertheless, LHON cells seem to be able to cope with the ETC dysfunction, maintaining apparently a normal growth and total cellular ATP level, yet being more vulnerable to metabolic/oxidative stress or other stressful conditions [2, 17].

Consistently, a recently developed mouse model revealed, together with some of the key histopathological features typically observed in LHON patients, the decrease of complex I activity, respiratory defects, and the increase in ROS levels, but no reduction in ATP synthesis, pointing to oxidative stress as the major driver of the pathology [18]. On these bases, most of therapeutic approaches to LHON, and generally to mitochondrial disorders, currently rely on the use of mitochondrial substrates, together with redox active effectors and free radical scavengers. Idebenone, curcumin, and vitamins C and E as well as other antioxidant compounds have been used separately or combined in “cocktails” customized for the individual patients [19, 20], all treatments unfortunately with limited success.

Under severe pathological conditions, such as inflammation or sepsis, a disruption of the homeostatic control of oxygen supply and utilization has been observed, accompanied by ROS formation, together with an imbalance of nitric oxide (NO) production and breakdown. Nitric oxide produced either enzymatically by nitric oxide synthases (NOSs) [21] or directly via the reduction of bulk nitrite at low pH [22] is a fundamental second messenger involved in a number of pathophysiological processes [23], inducing detrimental or cytoprotective effects depending on its concentration and localization [24]. Importantly, NO regulates mitochondrial respiration by reversibly binding to cytochrome *c* oxidase and limiting O_2_ consumption, while extending O_2_ gradients in tissues [25, 26]. Such inhibition is more effective in actively respiring cells and at low oxygen concentration [27–29]. Under conditions associated with increased ROS levels, NO participates in reactions with other reactive species to generate secondary products that can impair mitochondrial function. Particularly, the reaction of NO and superoxide anion (O_2_^−∙^), leading to the formation of toxic peroxynitrite (ONOO^−^), is very rapid (diffusion limited) and known to induce macromolecular damage, including nitration and inactivation of mitochondrial proteins [30].

Although many biochemical aspects of LHON have been elucidated, the role of NO in the LHON disease has not been investigated yet. Interestingly, an increased immunoreactivity for inducible nitric oxide synthase (iNOS) has been detected in macrophages and in the microglia of demyelinated lesions in the brain white matter of a LHON female patient, suggesting an early immunological mechanism in addition to the primary degeneration of the optic nerve [31]. In this patient, administration of corticosteroids improved visual and neurological function. This observation suggests that increased NO levels, as those produced by iNOS, in combination with ROS overproduction can result into mitochondrial defects, eventually triggering severe cell dysfunctions.

Here, we tested this hypothesis on PBMCs and lymphoblasts derived from a female LHON patient with bilateral reduced vision and immunological disorders. We found that the cells, although carrying the pathogenic mutation 11778/ND4, are still endowed with a suitable bioenergetic apparatus, producing ATP, thus well compensating complex I mutation. However, lymphoblasts proved to be more susceptible to NO toxicity, suggesting that the mitochondrial genetic defect, via the enhancement of the basal ROS levels, potentiates the formation of secondary reactive nitrogen species (RNS), particularly ONOO^−^, leading to reduced cell viability.

## 2. Materials and Methods

### 2.1. Chemicals

RPMI-1640 medium, fetal bovine serum (FBS), and nonessential amino acid solution were from Sigma-Aldrich and Invitrogen Life Technologies (GIBCO). Thiazolyl blue tetrazolium bromide (MTT) was from Sigma-Aldrich and (Z)-1-[-2-(aminoethyl)-N-(2-ammonioethyl)amino]diazen-1-ium-1,2-diolate (DETA-NONOate) was purchased from Cayman Chemicals.

### 2.2. Case Report

The proband is 34-year-old woman with a previous diagnosis of autoimmune thyroiditis. When she was 28 years old, she suffered a sudden and painful loss of vision in OS and after two weeks in OD, accompanied by xerophthalmia. Visual field showed retinal sensibility reduction, especially at superior areas, whereas fluoroangiography was normal. She was treated with high doses of corticosteroids and intravenous immunoglobulins without benefits. Brain MRI scan was unremarkable. After 4 months, plasmapheresis was performed with little improvement, so it was decided to start cyclophosphamide treatment. This treatment was carried on for 6 months without any benefit. In the meantime, after a femur fracture due to an accidental fall, a severe osteoporosis was diagnosed. After one year from the visual loss, a workout for autoimmune disorders gave positive ANA (speckled pattern, 1 : 160) and the presence of anti-Ro/SSA. Cytochemical, bacteriological, viral, and immunoelectrophoretic analyses on cerebrospinal fluid were normal.

Brainstem auditory evoked responses, along with motor and somatosensory evoked potentials, were normal, while visual evoked potentials could not be performed due to bilateral visual loss. A new brain and orbital and spinal cord MRI scan showed bilateral callosal, periventricular, and paratrigonal white matter symmetric hyperintensities on T2-weighted images without contrast enhancement on T1. No alterations were found at the level of the spinal cord and optic nerves. On admission to our neurological division, bilateral visual acuity was 1/10. We excluded neoplastic or paraneoplastic processes by full-body CT scan and screening for antibodies associated with paraneoplastic syndromes. The infectious aetiology was also discarded after negative hepatitis B and hepatitis C virus, HIV, EBV, cytomegalovirus serology, and tuberculin skin test. Neuromyelitis optica, carential, metabolic, and endocrinological disorders were also excluded. The standard genetic screening for LHON disease showed the presence of 11778/ND4 mutation. She began ubidecarenone therapy (600 mg/day) without any subjective or measurable improvement of visual acuity. After two months she developed* Escherichia coli* pyelonephritis and chronic tubulointerstitial nephritis was diagnosed. After one year and a half from the visual loss, the patient presented a bilateral visual acuity worsening with a severe headache and hypoesthesia at four limbs. A brain MRI showed a massive extension of bilateral paratrigonal white matter lesions with an involvement of bilateral occipital subcortical white matter and increased signal intensity on T2-weighted images of optical radiations and retrochiasmatic optic tracts. After one month, brain MRI with contrast and diffusion weighted imaging was repeated showing an additional enlargement of bilateral occipital lesions with positivity in DWI sequence and a circled enhancement. Therefore she was admitted again to our division and a hemogasanalysis proved a severe metabolic acidosis (pH 7.14, pCO_2_ = 13 mmHg, pO_2_ = 145 mmHg, sO_2_ = 98.5%, HCO_3_^−^ = 4.40 mmol/L, BE = −22.40 mmol/L, and BE_ecf_ = −24.60). Blood lactate measurements showed an abnormal increase during exercise (at rest 13.69 mg/dL; after 5-minute exercise 48.13 mg/dL; after 10-minute exercise 65.53 mg/dL; after 15-minute exercise 85.57 mg/dL; 15 minutes after the end of exercise 59.28 mg/dL; and normal value 4.5–19.8). Blood analysis showed a renal injury associated with nephrocalcinosis diagnosed by ultrasonography and CT scan. During the hospitalization the medical care focused on the improvement of electrolyte balance and renal injury until values normalization. Simultaneously, by brain MRI scan, we appreciated a clear reduction of white matter lesions and contrast enhancement absence.

### 2.3. Peripheral Blood Mononuclear Cells (PBMCs) Isolation

Blood samples were obtained from the case report and from three matched (age, sex, and geographical origin) healthy controls. The local Institutional Review Board approved the study and all participating subjects gave written informed consent.

PBMCs were obtained by density centrifugation over Ficoll-Hypaque according to standard procedures. Genomic DNA was extracted from PBMCs using a commercial kit (QIAamp DNA Mini Kit, Qiagen). Purified PBMCs (5 × 10^4^ cells/well) were seeded in 96-flat well plates and cultured in 200 *μ*L/well RPMI-1640 medium, supplemented with 20% FBS (HyClone), 2 mM L-glutamine, 100 U/mL penicillin, and 100 *μ*g/mL streptomycin.

### 2.4. Preparation and Cultures of Lymphoblast Cell Lines

Lymphoblast cell lines were established from peripheral blood PBMCs of the patient and the three healthy controls by EBV infection. The mutational analysis of the patient mtDNA was performed by PCR, restriction analysis, and electrophoresis and confirmed by sequencing (Sanger method). Both PBMCs and lymphoblast cell lines derived from the LHON patient and controls were grown in RPMI-1640 medium supplemented with 20% (v/v) heat-inactivated fetal bovine serum (FBS), 2 mM L-glutamine, 100 U/mL penicillin, 100 *μ*g/mL streptomycin, and 1X nonessential amino acids. Cells were cultured in 25 cm^2^ or 75 cm^2^ flasks or in multiwell plates and incubated at 37°C under standard conditions [5% CO_2_; 95% relative humidity]. Viable-cell counting was carried out using the Trypan blue dye exclusion test (Sigma-Aldrich).

### 2.5. Cell Respiration

Oxygen consumption in intact PBMCs derived from the LHON patient and controls was measured using a high resolution respirometer (2k-Oxygraph, OROBOROS Instruments). Data acquisition and analysis were carried out using the software Datalab (OROBOROS Instruments). Control and LHON PBMCs were collected, washed with sterile phosphate buffered saline (PBS), counted, and suspended in DMEM containing 1 g/L glucose. Cells, at a final density ranging from 7 to 9 × 10^6^ cells/mL, were incubated in the 2k-Oxygraph chambers at 37°C for 30 min to allow temperature and pH equilibration. Respiration was evaluated under basal metabolic conditions, thus sustained by endogenous substrates. After recording an oxygen consumption rate (OCR) baseline, 4 *μ*M antimycin A (AA) was added to the chamber in order to inhibit cytochrome *c* reductase (complex III) and stop mitochondrial respiration. Data were recorded at sampling intervals of 2 s.

### 2.6. Determination of ATP Levels

Cellular concentration of ATP was measured by chemiluminescence, under stationary conditions. Cells were incubated overnight in antibiotic/FBS-free DMEM medium. Steady-state ATP levels were measured in LHON and control PBMCs (3 × 10^5^ cells/mL) 4 h after resuspending the cells in PBS containing L-glutamine (2 mM), in the presence of glucose (2 g/L) or its absence to stimulate OXPHOS; when necessary, oligomycin (2.5 *μ*g/mL) was added over the last 1.5 h of incubation. Synthesized ATP was measured using the ATPlite 1step kit (PerkinElmer) in a luminometer (VICTOR Multilabel Counter, PerkinElmer, USA) equipped with 96-well plates.

### 2.7. Reactive Oxygen Species (ROS) Quantification by Flow Cytometry

To measure the intracellular ROS concentration, the fluorescent probe 2′,7′-dichlorofluorescein diacetate (DCFDA, Sigma-Aldrich) was used. PBMCs from the LHON patient and controls were incubated for 30 min at 37°C in the dark in RPMI-1640 medium containing 10 *μ*M DCFDA. Afterwards, cells were washed twice with Hank's Buffered Salt Solution (HBSS) supplemented with calcium and magnesium, as suggested by the manufacturer, collected, and analysed immediately by flow cytometry (BD Accuri C6); ROS levels were estimated from the mean fluorescence intensity. The green fluorescence was measured using the FL-1 setting (log mode) after having gated out cell debris. In each experiment 10.000 events were recorded.

### 2.8. 3-Nitrotyrosine Level Detection

The content in 3-nitrotyrosine (3-NT) modified proteins was used as marker of protein damage by ONOO^−^. The intracellular 3-NT levels were assessed colorimetrically using a competitive ELISA kit (Abcam). For each independent experiment a standard curve was generated with the provided 3-NT standard and the 3-NT content quantified.

### 2.9. Nitrate/Nitrite (NO_*x*_) Determination

NO_*x*_ concentration in cell supernatants was determined fluorimetrically using Fluorimetric Assay Kit (Cayman Chemical), 48 h after cells seeding.

### 2.10. Cell Viability Assay

After 48 h incubation in presence or absence of the NO donor DETA-NONOate, the viability of lymphoblast cells was assessed using the MTT [-3(4,5-dimethylthiazol-2-yl)-2,5-diphenyltetrazolium bromide] reduction assay as described in [32]. Briefly, cells (2 × 10^5^/mL) were seeded in a 96-well plate and incubated for 48 h at 37°C in the presence of increasing DETA-NONOate concentrations (0.01–0.5 mM) in a final volume of 100 *μ*L/well. Having a half-life of 20 h at 37°C, the NO donor was readded after 24 h. At the end of the 48 h incubation, 10 *μ*L of MTT solution (5 mg/mL) was added to each well, followed by 4 h incubation at 37°C. Afterwards, in order to dissolve the dark-coloured formazan crystals produced by reduction of the MTT tetrazolium salt, cells were incubated at 37°C overnight with 100 *μ*L of 10% sodium dodecyl sulphate (SDS) in 0.01 M HCl. The optical density of reduced MTT was measured at 570 nm with a reference wavelength at 690 nm using Appliskan Microplate Reader (Thermo Scientific). The experiments were carried out in triplicate.

### 2.11. Statistical Analysis

Data are reported as mean ± SEM of at least three independent experiments and significance (*P*) was determined using Student's *t*-test. *P* values ≤ 0.05 were considered significant.

## 3. Results

### 3.1. Mitochondrial Function in LHON Patient PBMCs

**Figure 1:**
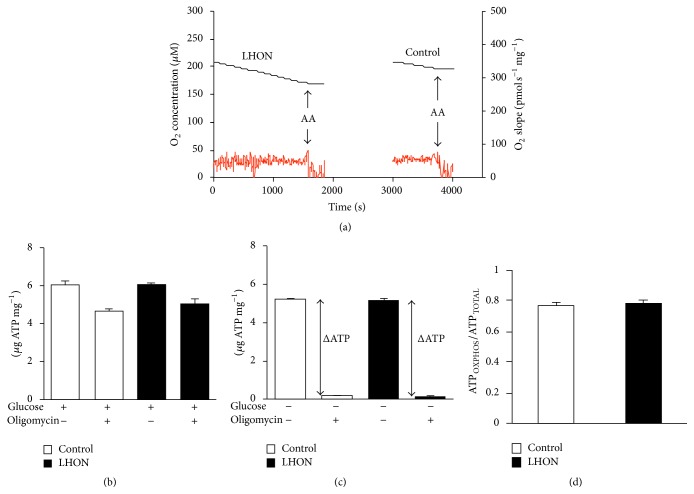
Bioenergetic properties of 11778/ND4 mutated PBMCs. (a) Representative cell O_2_ consumption measurements. Black line: O_2_ concentration trace; red line: O_2_ consumption rate. After recording basal respiration, 4 *μ*M antimycin A (AA) was added. (b) Cellular ATP levels measured in the presence of glucose with and without oligomycin. (c) Cellular ATP levels measured in the absence of glucose with and without oligomycin. (d) Fractional ATP expressed as the ratio between ATP_OXPHOS_ and ATP_TOTAL_.   ATP_OXPHOS_ was obtained from ΔATP (c) and ATP_TOTAL_ was measured in the presence of glucose without oligomycin (b). Data (mean ± SEM) were collected in three triplicate experiments on cells derived from the LHON patient and the three healthy controls.

RGCs, brain, and kidneys, the most affected sites in the LHON patient, are all high-energy demanding, so they rely more on OXPHOS ATP. Importantly, PBMCs have a metabolism mainly sustained by OXPHOS [33, 34] and therefore represent a good model to be investigated in the present study.  Figure 1(a) shows a typical oxygen consumption trace acquired with intact PBMCs. The oxygen consumption was sustained by endogenous substrates and relied almost completely on mitochondria, as it was almost completely inhibited by antimycin A, a specific inhibitor of cytochrome *c* reductase. After normalization to the protein content, we found the O_2_ consumption rate (≈50 pmol O_2_ s^−1^ mg^−1^) to be very similar in both LHON and control cells.

In agreement with these data, no significant differences in the steady-state concentration of ATP were observed in control and patient PBMCs. ATP levels were evaluated either in the absence or in the presence of glucose to sustain glycolysis and using oligomycin to inhibit OXPHOS activity. When glucose was present (Figure 1(b)), both LHON and control cells displayed a similar ATP content (ATP_TOTAL_ ≈ 6 *μ*g ATP/mg total protein), and oligomycin inhibition of OXPHOS was found poorly effective in control and LHON PBMCs, suggesting that both cell types possess an efficient glucose-dependent glycolytic compensation (Warburg effect). Without glucose (Figure 1(c)), both LHON and control cells showed ~20% decrease in the ATP content, whose concentration dropped dramatically in the presence of oligomycin. Under the latter conditions, the glycolytic contribution is minimal and the difference between the ATP measured in the presence and absence of oligomycin (ΔATP) is indicative of the ATP generated by OXPHOS (ATP_OXPHOS_). The ATP_OXPHOS_/ATP_TOTAL_ ratio (Figure 1(d)) is very similar in LHON and control cells, indicating that the mutation does not affect OXPHOS efficiency.

Taken together, these results suggest that, over and above a bioenergetic deficit imputable to complex I mutation, other molecular mechanisms contribute to the clinical onset of LHON and its progress that we hypothesize to be also related to increased cell levels of reactive oxygen and nitrogen species.

### 3.2. Increased Level of ROS, 3-Nitrotyrosine, and Nitrite in PBMCs

**Figure 2:**
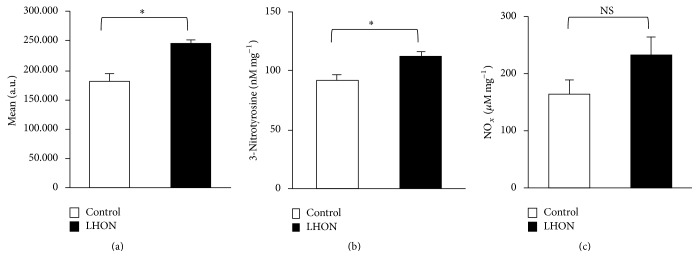
Oxidative and nitrosative stress is increased in 11778/ND4 mutated PBMCs. (a) Intracellular ROS levels were measured using the DCFDA dye. (b) The 3-NT content was determined by competitive ELISA and normalized to total protein. (c) Concentration of NO_*x*_ (nitrite and nitrate) in the cell supernatant as normalized to total protein. Data (mean ± SEM) collected in three duplicate experiments on cells derived from the LHON patient and the three healthy controls. Values are considered significant when *P* < 0.05: ^*∗*^ *P* ≤ 0.05; Ns: not significant.

In order to investigate whether the PBMCs derived from the LHON patient displayed increased oxidative/nitrosative stress levels, we measured the amount of ROS and 3-NT under basal conditions. The latter is an important marker of RNS, including ONOO^−^. We found that LHON PBMCs display a significant 1.4-fold increase in ROS production compared to controls (Figure 2(a)). Consistently, LHON PBMCs displayed a higher concentration of 3-NT, suggesting a role of NO chemistry in cellular stress (Figure 2(b)). To assess the basal NO level in LHON, we measured the concentration of nitrite/nitrate, the oxidation products of NO. Interestingly we found that the nitrite/nitrate levels (Figure 2(c)) tend to increase compared to controls, although the reported variation did not reach statistical significance.

Owing to a limited availability of biological samples (cells from patient) and in order to obtain larger amounts of starting material, EBV-transformed lymphoblasts were used for further cell biochemical analysis. The bioenergetic behaviour of primary and transformed cells was very similar, with the two cell types displaying superimposable mitochondrial parameters (data not shown).

### 3.3. DETA-NONOate Decreases LHON Lymphoblasts Viability

**Figure 3:**
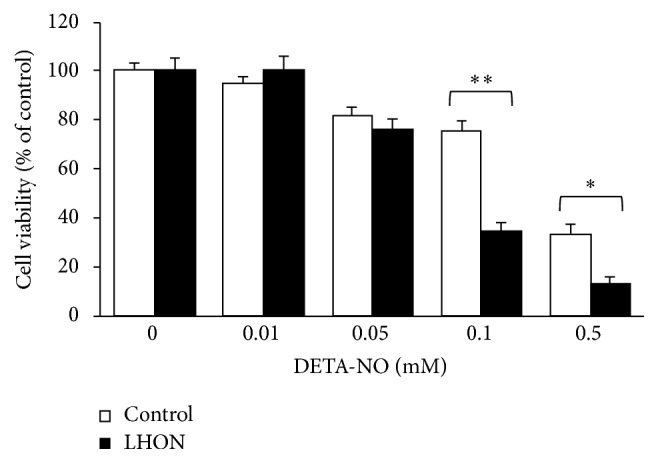
NO impairs the viability of 11778/ND4 mutated lymphoblasts. The cells derived from the LHON patient and the three healthy controls were treated with various concentrations of DETA-NONOate for 48 h and their viability was assessed using the MTT assay. Data are shown as the percentage of cell viability measured in the absence of the NO releaser. Data acquired in three triplicate experiments are expressed as mean ± SEM. Values are considered significant when *P* < 0.05: ^*∗*^ *P* ≤ 0.05; ^*∗∗*^ *P* ≤ 0.01.

A chronic increase in NO levels was artificially mimicked using the NO donor DETA-NONOate (DETA-NO). Cell viability was examined in both LHON and control lymphoblasts after 48 h incubation with different amounts of the NO releaser. As a regulator of cell proliferation, NO can either enhance or inhibit cell growth depending on its concentration.  Figure 3 shows that at 10 *μ*M DETA-NO no cytotoxicity was associated with NO exposure. Higher concentrations of DETA-NO affected cell viability proportionally to the amount added. However, the decline in cell viability was significantly larger in LHON than in control cells: at 0.1 and 0.5 mM DETA-NO, the residual viability of LHON cells was 35% and 15%, respectively, while in control cells it was, respectively, 75% and 35%. Trypan blue exclusion assays, carried out under comparable experimental conditions, indicated a similar cellular behaviour (data not shown), showing that LHON cells are more susceptible to nitrosative stress than control cells.

## 4. Discussion

LHON is characterized by a low penetrance, as most individuals carrying mtDNA mutations remain asymptomatic, with a limited subset of them expressing the disease. Age and gender are the most important risk factors, with approximately 30–50% of males and 10–20% of female LHON mutation carriers becoming symptomatic at a median age of 19 years (range 5–56 years) [35]. Indeed, genetic, epigenetic, and environmental factors all contribute to the onset and evolution of the disease [36]. Complex I mutation, the primary etiologic cause of LHON, despite being a necessary determinant of the pathology, is not sufficient to induce the clinical expression of the disease so that a convincing explanation for the pathogenesis of this disorder is still under debate. The impairment of bioenergetics parameters, such as cell respiration and ATP production, has been found absolutely modest both in patient's tissues and in cell models of the disease. On the contrary, a marked overproduction of ROS has been reported, together with a low capability of the cellular antioxidant machinery to maintain the redox homeostasis.

LHON cybrid models have shown lower levels of glutathione peroxidase, glutathione reductase, and Mn-superoxide dismutase (MnSOD) or total glutathione, compared to controls [37, 38], particularly after cell treatment to enhance their OXPHOS dependency. These observations strongly support the idea that the genetic alteration of complex I, over and above the simple action on primary mitochondrial parameters, induces an alteration of the cell redox-signalling and homeostasis. Interestingly, in a cybrid model carrying the G11778A/ND4 mutation, it has been observed that mitochondrial overexpression of MnSOD induces a decrease in superoxide levels and enhances cell survival [39]. While chronic oxidative stress has been shown to play an important role in the onset of the disease, the role of NO and related reactive species remained unexplored. Together with CO and H_2_S, NO forms the gasotransmitter triad acting as a redox-signalling regulator of several physiological functions, the mitochondrial one included [40]. NO, particularly at low concentrations, protects against cell death [41–44], whereas in the presence of superoxide it becomes toxic by forming ONOO^−^ [30]. It should be kept in mind, however, that, even in the absence of ONOO^−^, depending on the electron flux level through the respiratory chain, that is, on the concentration of mitochondrial ferrocytochrome *c*, and particularly under low O_2_ tension (hypoxia), inhibition of mitochondrial complex IV by NO can be severe and persistent [29, 45, 46].

According to our results, LHON cells are apparently more prone to such NO-dependent detrimental chemistry. We present here the case of a female patient with a point mutation at nucleotide position G11778A, who suffered from both eyes' visual loss and subsequently displayed markers for autoimmune disorders, as well as white matter alterations outside the visual system.

We show that PBMCs and lymphoblasts derived from our patient are more susceptible not only to oxidative but also to nitrosative stress. In these cells we found that, under metabolic basal conditions and in the absence of exogenous NO, the 3-NT levels are enhanced. This finding is in agreement with prior studies carried out on optic nerve and retinal histological specimens obtained from LHON patients and on synaptosomes from a mouse model of the disease that have evidenced increased levels of 3-NT [18, 47]. The higher basal 3-NT concentration suggests that LHON cells produce larger amounts of nitrosating agents, such as ONOO^−^. Consistently, we found that the concentration of nitrite/nitrate, the oxidation products of NO, tends to increase in patient cells, compared to controls. Increase in nitrosative stress might be particularly likely when NO overproduction takes place.

The current study reveals that chronic exposure to NO in proband lymphoblasts carrying the 11778/ND4 mutation determines a significant decrease in cell viability compared to controls, suggesting that in this pathological state the NO chemistry is more active. Interestingly, our patient has shown an abnormal increase in lactate serum levels after exercise, suggesting a limited mitochondrial reserve capacity. In terms of energy metabolism, under basal conditions the patient PBMCs are able to fully compensate complex I mutation allowing an efficient mitochondrial function. We observed that the ATP levels in LHON cells were not significantly different from those ones of control cells, even when the glycolytic system was restricted by glucose deprivation. Both cell types were equally able to efficiently compensate OXPHOS defects with glycolysis, as shown by abolishing the contribution of ATP_OXPHOS_ with oligomycin.

Mitochondrial ATP generation is crucial for cell function; therefore mitochondria have evolved several different strategies to maintain it to face dysfunctions. Switching to glycolysis is fundamental for cell survival during acute stress, although this pathway represents a much less efficient mode of ATP production, resulting in an energy deficit and acidosis over long periods, especially in those cells relying on high-energy demand. Cells derived from LHON carriers, having the mitochondrial mutation but not expressing the disease, were shown to adopt different responses that allow the maintenance of the respiratory function and ATP levels, such as increasing the activity of the succinate-dependent pathway or the mitochondrial mass [13, 48]. The bioenergetic compensation observed in individuals affected by LHON can be disrupted by various factors, such as the frequently suggested nuclear modifying genes and environmental triggers. Smoking and alcohol are both known to decrease the mitochondrial reserve capacity [49–51] and have been reported to play a role in the onset of LHON disease [36] also contributing to its incomplete penetrance. A loss of mitochondrial reserve capacity, as obtained by the chronic exposure to NO, decreases the ability to respond to endogenous secondary energetic stressors, such as ROS and RNS, which we observed to be increased in the patient PBMCs. All together these findings point to a novel role played by RNS, particularly ONOO^−^, whose accumulation in LHON cells appears to have a role in the pathology and its pharmacological control may be important in the patients [52].

## 5. Conclusions

In conclusion, we show that lymphoblasts derived from a LHON patient carrying the 11788/ND4 mutation are more susceptible to NO, suggesting that the exposure to high NO concentrations could impair* in vivo* the ability to cope with the oxidative stress caused by the genetic defect, thereby driving the pathology. This unprecedented observation is consistent with both LHON cell and animal models pointing to oxidative stress and impairment of redox homeostasis as important factors contributing to the onset and progression of the disease. Further work is needed to extend these studies to other LHON cells or tissues in order to better understand the reciprocal role of ROS and RNS in this pathology and to design new drugs and appropriate therapeutic approaches.
